# Facial biotype classification for orthodontic treatment planning using an alternative learning algorithm for tree augmented Naive Bayes

**DOI:** 10.1186/s12911-022-02062-7

**Published:** 2022-12-01

**Authors:** Gonzalo A. Ruz, Pamela Araya-Díaz, Pablo A. Henríquez

**Affiliations:** 1 Facultad de Ingeniería y Ciencias, Universidad Adolfo Ibáñez, Santiago, Chile; 2 Center of Applied Ecology and Sustainability (CAPES), Santiago, Chile; 3 Data Observatory Foundation, Santiago, Chile; 4 Departamento del Niño y Adolescente, Área de Ortodoncia, Facultad de Odontología, Universidad Andres Bello, Santiago, Chile; 5 Facultad de Administración y Economía, Universidad Diego Portales, Santiago, Chile

**Keywords:** Bayesian networks, Tree augmented Naive Bayes, Evolution strategy, Facial biotypes, Orthodontic treatment planning

## Abstract

**Background:**

When designing a treatment in orthodontics, especially for children and teenagers, it is crucial to be aware of the changes that occur throughout facial growth because the rate and direction of growth can greatly affect the necessity of using different treatment mechanics. This paper presents a Bayesian network approach for facial biotype classification to classify patients’ biotypes into Dolichofacial (long and narrow face), Brachyfacial (short and wide face), and an intermediate kind called Mesofacial, we develop a novel learning technique for tree augmented Naive Bayes (TAN) for this purpose.

**Results:**

The proposed method, on average, outperformed all the other models based on accuracy, precision, recall, $F_{1}\hbox {-score}$, and kappa, for the particular dataset analyzed. Moreover, the proposed method presented the lowest dispersion, making this model more stable and robust against different runs.

**Conclusions:**

The proposed method obtained high accuracy values compared to other competitive classifiers. When analyzing a resulting Bayesian network, many of the interactions shown in the network had an orthodontic interpretation. For orthodontists, the Bayesian network classifier can be a helpful decision-making tool.

## Background

In recent years, there has been a rise in the use of machine learning-based tools in medical treatments to aid in decision-making for treatment planning. In particular, the output of these models can be used as a support tool for health personnel who ultimately make decisions. Given the implications for patients on these decisions, the machine learning technique used should be interpretable. An interesting machine learning technique for this purpose is Bayesian networks (BN) [1], which combines graph theory with probability theory.

In the field of dentistry BN have been applied in diverse areas. For example, in [2] prior to and during the application of a certain orthodontic procedure, BN were employed to describe certain tooth color parameters. To better understand the underlying data structure of the patterns of dental caries in the population, the prevalence of dental caries in the primary dentition of 352 Myanmar schoolchildren was examined at the tooth level using BN in [3]. The effectiveness of BN in the assessment of dental age-related evidence obtained using a geometrical approximation approach of the pulp chamber volume was examined in [4]. BN are used in [5] for age estimation and classification based on dental evidence, in particular, to the development of third molars. A BN clinical decision support system was designed in [6] to assist general practitioners in determining whether patients with permanent dentition need orthodontic treatment. A Dental Caries Clinical Decision Support System is evaluated in [7] which uses a BN to provide suggestions and represent clinical patterns. The outcomes demonstrated the Bayesian network’s accuracy in various cases. In [8], a minimally invasive method for elevating the lateral maxillary sinus was described, and BN was used to determine the link between the parameters involved. The use of BN to MR images to identify temporomandibular disorders was looked at in [9]. The goal was to ascertain how temporomandibular disorders were diagnosed, concentrating on how each discovery affected the other. The findings demonstrated that the BN path condition method was more than 99% accurate when employing resubstitution validation and 10-fold cross-validation. The key benefit of utilizing BN, however, is its ability to express the causal links between various data and assign conditional probabilities, which might subsequently be utilized to interpret the course of temporomandibular disorders. In [10], BN are used to identify and depict the relationships between several Class III malocclusion maxillofacial features during growth and treatment. The authors demonstrate that as compared to individuals undergoing orthodontic treatment with rapid maxillary expansion and facemask therapy, untreated participants exhibit different Class III craniofacial growth patterns. Also, it is important to point out that BN have been used for meta-analysis in several dental research topics [11–16].

BN are probabilistic graphical models representing discrete random variables and conditional dependencies via a directed acyclic graph (DAG). In classification (supervised learning) problems, when using a probabilistic approach, the difficulty is to compute effectively the posterior probability of the class variable $Y_{k}$ (with $k=1,\dots ,K$) given an *n*-dimensional input data point ${\mathbf {x}}=(x_{1},\dots ,x_{n})$. This can be carried out using the Bayes rule:

1 $$\begin{aligned} p(Y_{k}|{\mathbf {x}})=\frac{p(Y_{k})p({\mathbf {x}}|Y_{k})}{p({\mathbf {x}})}. \end{aligned}$$

The numerator, which comprises the a priori probability of the class variable and the *likelihood* (the joint probability of the input features conditioned to the class variable), is what is important in this case. The calculation of the class variable’s a priori probability is simple. It can be determined from the training set’s class variable values’ relative frequency. However, there are numerous methods for calculating *likelihood*. The usage of Bayesian networks, thus, Bayesian network classifiers [17], is one of them.

There are various Bayesian network classifiers [18–23]. However, the two most often used are the tree augmented Naive Bayes (TAN) classifier [17] and the Naive Bayesian network classifier, also known as the Naive Bayes [24]. The Naive Bayes approach computes the likelihood in (1) by assuming conditional independence among the attributes given the class variable. There are no edges between the attributes as a result. As opposed to TAN, which begins by taking into account a fully connected network with weighted edges, it uses the *conditional mutual information* between pairs of attributes to generate these weights. Then, the application of Kruskal’s algorithm (the maximum weighted spanning tree (MWST)) to produce a tree structure is carried out, leaving just $n-1$ edges. Each attribute in this version of the Bayesian network classifier will have an incoming edge from another attribute, with the exception of the selected root attribute node.

The TAN model corrects the naive version’s strong assumption of conditional independence. Theoretically, it ought to deliver better outcomes (accuracy) than the Naive Bayes. However, TAN has significant drawbacks, one of which is its difficulty to estimate the conditional mutual information accurately. Two direct difficulties when working with conditional mutual information are: (1) the computational complexity for *n* nodes and *N* training samples is ${\mathcal {O}}(n^{2}N)$ [25], therefore, for datasets with many attributes the computation becomes very slow, needing more computational power, (2) the conditional mutual information estimate produced when there are not enough training instances in each class to accurately estimate the joint probability distribution and the conditional distributions. This is significant because conditional mutual information is used as weights in the fully connected graph throughout TAN’s tree construction technique. The obvious question is: Can the network weights be learned from the data to achieve satisfactory classification results without estimating conditional mutual information?

When preparing a treatment in orthodontics, especially for children and teenagers, it is crucial to be aware of the changes that take place throughout facial growth because the rate and direction of growth can greatly affect the necessity of using different treatment mechanics. The Ricketts’ VERT index is one of the most widely used methods for identifying facial biotypes [26]. The biotypes can be divided into Dolichofacial (long and narrow face), Brachyfacial (short and wide face), and an intermediate form known as Mesofacial based on the VERT index.

In this paper, we propose a different approach for learning TAN classifiers without estimating conditional mutual information. Instead, we use an evolution strategy to learn the weights of the networks from the data. Using attributes that are unaffected by the sagittal position of the jaws, we apply the proposed method to automatically classify a patient’s biotype, eradicating the inaccuracies shown with the VERT index. In particular, one of the measurements used to calculate the VERT index is the facial depth, which indicates the sagittal relationship between the jaws. When this sagittal relationship is altered, the VERT is also altered. Therefore, a higher VERT is obtained in individuals with a prominent jaw, diagnosing the patient as more Brachyfacial than it is. Conversely, a patient with a mandible positioned further back will appear more Dolichofacial than it is.

## Results

The results are shown in Table 1. Overall we notice that $(\mu ,\lambda )$-TAN, on average, outperforms all the other models for the particular dataset analyzed. Moreover, $(\mu ,\lambda )$-TAN presents the lowest dispersion, making this model more stable and robust against different runs.

Table 2 shows that the results in terms of Accuracy of $(\mu ,\lambda )$-TAN are statistically significantly different to the results obtained by the other methods. Also, it is important to highlight that the results obtained are better than previously published results for the same dataset [27].

**Fig. 1:**
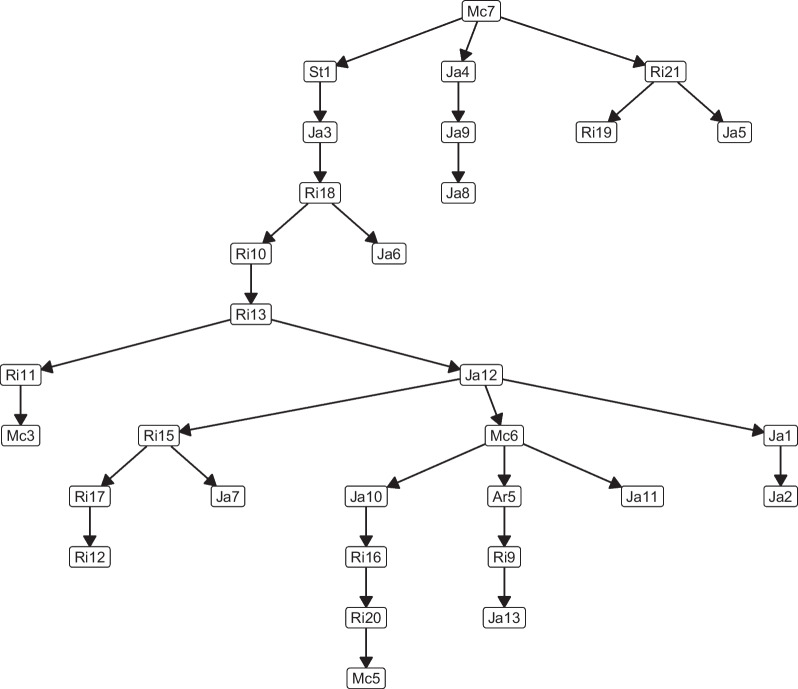
Shows the best $(\mu ,\lambda )$-TAN model obtained throughout the 20 runs. The $(\mu ,\lambda )$-TAN classifier for the facial biotype dataset

The best resulting network using $(\mu ,\lambda )$-TAN is shown in Fig. 1. For better visualization, we have omitted in this figure, the node with the class variable and the edges from this node to all the other nodes.

**Fig. 2:**
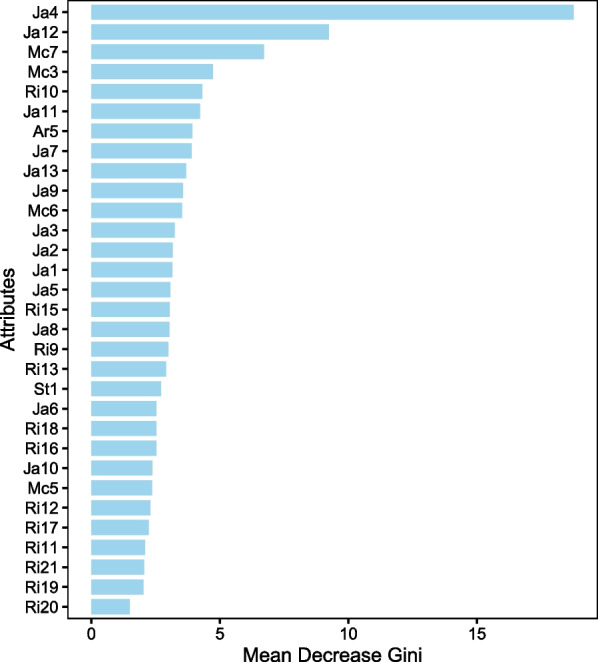
Attribute importance. Attributes ranking based on the Gini importance measure.

We used the *importance* function from the randomForest package in R [28] to create a smaller network. Based on the Gini importance, a metric used to assess the node impurity during the tree inference process, this function calculates the importance of each attribute (in decision trees or random forests). The outcome is displayed in Fig. 2.

**Table 1:** Performance measures for each model

Algorithm	Accuracy	Precision	Recall	$F_{1}$-score	Kappa
	Avg. ± SD.	Avg. ± SD.	Avg. ± SD.	Avg. ± SD.	Avg. ± SD.
NB	70.27 ± 5.21	70.30 ± 5.92	74.09 ± 6.66	70.72 ± 5.55	0.54 ± 0.11
TAN	71.01 ± 4.19	70.29 ± 5.84	74.21 ± 4.52	70.81 ± 5.39	0.56 ± 0.11
SVM	70.63 ± 4.43	70.31 ± 5.22	73.68 ± 5.26	70.55 ± 5.31	0.55 ± 0.08
DT	69.27 ± 7.19	69.93 ± 5.02	73.16 ± 4.14	70.72 ± 4.82	0.52 ± 0.10
RF	69.07 ± 4.93	67.70 ± 4.78	71.08 ± 7.41	67.30 ± 5.78	0.51 ± 0.07
RVFL	70.11 ± 5.34	70.44 ± 4.31	74.16 ± 4.32	71.19 ± 4.35	0.54 ± 0.11
ATAN	71.10 ± 5.77	70.22 ± 3.56	73.89 ± 4.29	71.36 ± 4.36	0.56 ± 0.09
HC-TAN	70.41 ± 7.44	69.67 ± 6.39	73.95 ± 6.04	70.22 ± 6.26	0.58 ± 0.11
HC-SP-TAN	70.81 ± 6.48	71.63 ± 4.81	74.98 ± 5.98	71.98 ± 5.36	0.56 ± 0.11
BSEJ	71.09 ± 4.24	72.09 ± 5.95	74.28 ± 5.22	71.09 ± 5.11	0.55 ± 0.12
FSSJ	71.69 ± 3.92	72.03 ± 3.34	73.88 ± 5.02	72.27 ± 4.56	0.58 ± 0.09
$(\mu ,\lambda )$-TAN	74.09 ± 3.62	73.89 ± 2.54	76.88 ± 2.34	75.14 ± 3.24	0.59 ± 0.08

**Table 2:** Statistical significance test for different simulations in terms of Accuracy

Algorithm	NB	TAN	SVM	DT	RF	RVFL	ATAN	HC-TAN	HC-SP-TAN	BSEJ	FSSJ
$(\mu ,\lambda )$-TAN	$\checkmark$	$\checkmark$	$\checkmark$	$\checkmark$	$\checkmark$	$\checkmark$	$\checkmark$	$\checkmark$	$\checkmark$	$\checkmark$	$\checkmark$

The $\checkmark$ symbol denotes that these two methods are statistically significantly different with $p<0.05$

Using the top four attributes from Fig. 2, the outcomes of our repeated experiments are displayed in Tables 3 and 4. We notice similar results as before, with slight improvements in the evaluation measures.

**Fig. 3:**
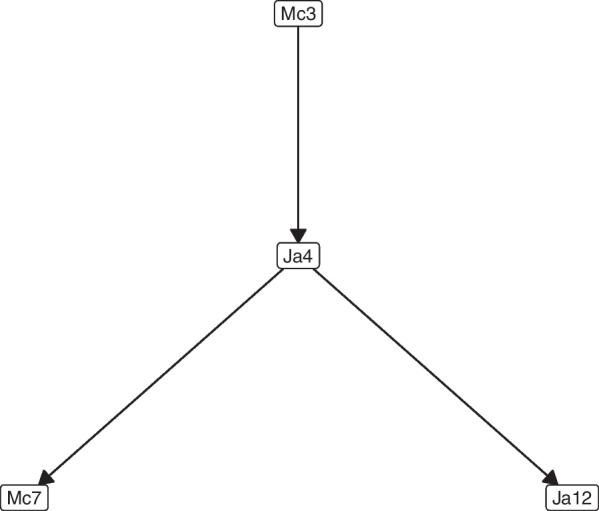
Shows the best $(\mu ,\lambda )$-TAN model obtained throughout the 20 runs. The $(\mu ,\lambda )$-TAN classifier for the facial biotype dataset (with the top 4 attributes)

The best resulting network in this case using $(\mu ,\lambda )$-TAN is shown in Fig. 3.

**Table 3:** Performance measures for each model (with four attributes)

Algorithm	Accuracy	Precision	Recall	$F_{1}$-score	Kappa
	Avg. ± SD.	Avg. ± SD.	Avg. ± SD.	Avg. ± SD.	Avg. ± SD.
NB	72.27 ± 5.03	72.36 ± 5.63	76.23 ± 5.61	72.84 ± 5.81	0.57 ± 0.09
TAN	72.18 ± 3.53	72.41 ± 6.66	76.43 ± 6.16	73.33 ± 6.95	0.56 ± 0.05
SVM	66.90 ± 4.59	67.86 ± 4.91	71.89 ± 6.22	69.23 ± 6.73	0.49 ± 0.07
DT	69.63 ± 5.21	69.63 ± 3.27	72.79 ± 2.74	70.04 ± 3.18	0.52 ± 0.08
RF	70.27 ± 4.06	71.28 ± 4.45	73.82 ± 4.24	71.11 ± 4.46	0.54 ± 0.06
RVFL	71.53 ± 5.67	72.51 ± 4.56	76.56 ± 6.21	73.11 ± 5.55	0.56 ± 0.09
ATAN	72.21 ± 4.78	72.25 ± 4.96	76.43 ± 6.78	73.01 ± 5.99	0.58 ± 0.09
HC-TAN	72.12 ± 5.01	72.07 ± 5.73	75.33 ± 3.71	72.73 ± 5.11	0.58 ± 0.10
HC-SP-TAN	71.09 ± 7.03	71.82 ± 5.34	74.27 ± 5.01	72.11 ± 5.28	0.55 ± 0.09
BSEJ	71.63 ± 6.03	71.76 ± 4.65	74.80 ± 5.12	72.26 ± 4.51	0.56 ± 0.09
FSSJ	72.27 ± 4.43	72.35 ± 4.75	76.20 ± 6.86	72.81 ± 6.26	0.58 ± 0.09
$(\mu ,\lambda )$-TAN	75.05 ± 3.86	74.85 ± 4.58	77.85 ± 4.46	75.51 ± 6.36	0.60 ± 0.07

**Table 4:** Statistical significance test for different simulations in terms of Accuracy

Algorithm	NB	TAN	SVM	DT	RF	RVFL	ATAN	HC-TAN	HC-SP-TAN	BSEJ	FSSJ
$(\mu ,\lambda )$-TAN	$\checkmark$	$\checkmark$	$\checkmark$	$\checkmark$	$\checkmark$	$\checkmark$	$\checkmark$	$\checkmark$	$\checkmark$	$\checkmark$	$\checkmark$

The $\checkmark$ symbol denotes that these two methods are statistically significantly different with $p<0.05$ (with four attributes)

To evaluate the robustness of the proposed method, we tested $(\mu ,\lambda )$-TAN on high-dimensional datasets chosen from the UCI database [29]. For this, three datasets were considered, as described in Table 5. Table 6 shows the performance of $(\mu ,\lambda )$-TAN and RF. It can be noticed that, in the case of the three datasets, our method achieves better performances on average.

**Table 5:** Information of the high-dimensional datasets

Datasets	Training	Testing	Attributes	Classes
Parkinson’s disease	529	227	754	2
Diabetes 130-US hospitals	71236	30530	50	2
Digital colposcopies	67	30	69	2

**Table 6:** Performance measures for proposed method using high-dimensional data

Algorithm	Datasets	Accuracy	Precision	Recall	$F_{1}$-score	Kappa
		Avg. ± SD.	Avg. ± SD.	Avg. ± SD.	Avg. ± SD.	Avg. ± SD.
RF	Parkinson’s disease	83.87 ± 1.39	74.58 ± 3.05	90.49 ± 3.97	81.72 ± 2.66	0.67 ± 0.05
$(\mu ,\lambda )$-TAN	Parkinson’s disease	85.02 ± 2.81	77.56 ± 4.12	90.26 ± 2.63	83.37 ± 2.79	0.69 ± 0.05
RF	Diabetes	87.03 ± 1.08	79.03 ± 4.11	91.90 ± 2.90	85.34 ± 3.09	0.73 ± 0.05
$(\mu ,\lambda )$-TAN	Diabetes	88.28 ± 3.42	81.36 ± 6.34	92.92 ± 5.24	86.54 ± 3.81	0.76 ± 0.06
RF	Digital colposcopies	84.33 ± 5.88	75.77 ± 2.56	90.23 ± 3.12	82.56 ± 2.99	0.68 ± 0.05
$(\mu ,\lambda )$-TAN	Digital colposcopies	85.91 ± 2.99	78.12 ± 3.45	90.88 ± 2.55	83.96 ± 2.81	0.69 ± 0.06

## Discussion

From Fig. 1 we notice that Mc7 (Lower anterior facial height) is the parent node of 3 variables, St1 (SNA angle), Ja4 (Lower Gonial angle), and Ri21 (Symphysis length). Mc7 is measured from a point close (anterior nasal spine) to one of the points that constitute the SNA angle (point A) and both points are part of the same structure (maxillary), so the modification of the first one could be accompanied of a modification of St1 as well. On the other hand, the relationship between Mc7, Ja4, and Ri21 is explained given that the three correspond to vertical measurements and the modification of one should be accompanied by the modification of the other two variables.

Ja4 is the parent node of Ja9 (Cranial base and Mandibular length ratio), a relationship for which we do not have a satisfactory biological explanation since Ja4 is a vertical measurement and Ja9 is a horizontal one. In turn, Ja9 is the parent node of Ja8 (Mandibular corpus length), which is explained given that Ja8 is one of the measurements that make up Ja9.

Ri21 is the parent node of Ri19 (Condylar height) and Ja5 (Anterior cranial base length), a relationship that does not have an acceptable biological explanation, except that, as they correspond to linear measurements, they are influenced by the volumetric proportionality that exists between the structures given the greater or lesser general size of the skull.

St1 is the parent node of Ja3 (Upper Gonial angle), a relationship that could be explained since both represent sagittal growth, St1 indicates sagittal position of the maxilla with respect to the skull and Ja3 horizontal projection of the mandible; Normally, both structures tend to grow proportionally in the sagittal direction. Ja3 is the parent node of Ri18 (Posterior height), which is explained by the fact that both measurements share a reference point (gonion). Ri18 is the parent node of Ri10 (Maxillary depth angle) and Ja6 (Posterior cranial base length), there is no biological explanation for the relationship between Ri18 and Ri10 since one corresponds to a sagittal measurement and the other is vertical and they are measured in different areas of the face. In the case of Ri18 and Ja6 they use different landmarks but both measure posterior height of the face, so a relationship between both variables is clearly explained.

Ri10 is the parent node of Ri13 (Anterior Cranial length), a relationship that can be explained since both measurements share a reference point (Nasion).

Ri13 is the parent node of Ri11 (Palatal plane angle) and Ja12 (Jarabak’s ratio), Ri13 and Ja12 share a reference point (Nasion) and all three correspond to vertical measurements, so the relationship between them is justifiable.

Ri11 is the parent node of Mc3 (Linear distance from point A to nasion perpendicular), which is explained because they share a reference point in the maxilla (Nasion) and the modification of this point would produce a change in both variables.

Ja12 (Jarabak’s ratio) is the parent node of 3 variables Ri15 (Mandibular corpus axis), Mc6 (Maxillary length), and Ja1 (Saddle angle). Regarding this relationship, Ja12 and Ri15 correspond to measures indicative of the magnitude of vertical growth, Ja1 is part of Ja12, and with Mc6 instead, it cannot be explained biologically in a satisfactory way.

Ja1 (Saddle angle) is the parent node of Ja2 (Articular angle), which is explained given that both are contiguous angles that tend to compensate each other, that is, the tendency is that if one angle increases, the other tends to decrease in post of maintaining the proportionality of the face.

Ri15 (Mandibular corpus axis) is the parent node of Ja7 (Ramus height) and Ri17 (Mandibular ramus position), a relationship that is explained by the fact that Ri15 and Ri17 share a reference point (Xi), and that the three measurements correspond to vertical variables.

Ri17 is the parent node of Ri12 (Cranial deflection), a relationship that is explained by the fact that both measurements contain the Porion-Orbitale line.

Mc6 (Maxillary length) is the parent node of Ja10 (Posterior facial height), Ar5 (Nasolabial angle), and Ja11 (Anterior facial height), a relationship that does not have an acceptable biological explanation, except for the volumetric proportionality that exists between the structures that contain the landmarks corresponding to MC6, Ja10, and Ja11, given the greater or lesser general size of the skull and that Ar5 can be influenced by Mc6 since the upper lip rests on the maxilla, although this relationship is not direct since it depends mainly on the sagittal position of the maxilla. Ar5 is the parent node of Ri9 (Maxillary height angle), which could be explained by the fact that, as in the previous case, the position of the upper lip can be modified given the position of the maxilla, although this relationship is not direct since Ri9 corresponds to a indicative measure of the vertical and not sagittal position of the maxilla. Ri9 is in turn the parent node of Ja13 (posterior cranial base ratio to ramus height), a relationship for which we do not have a satisfactory biological explanation since, although both are vertical measurements, they correspond to different areas of the face.

Ja10 (Posterior facial height) is the parent node of Ri16 (Articular cavity position: Porion to Ptv), however, there is no direct biological explanation for this relationship; Ri16 is in turn the parent node of Ri20 (Condylar neck length), although the condyle is in relation to the joint cavity, we did not find an explanation for the relationship between the sagittal position of the joint cavity (Ri16) and the length of the neck of the condyle; Ri20 is the parent node of Mc5 (mandibular length), a relationship that could be explained by the fact that Mc5 has a reference point in the condyle and this is related to the joint cavity and both are sagittal measurements.

When analyzing the importance of each variable shown in Fig. 2, we notice that the four variables that turned out to have the greatest discriminatory power are: Ja4 (Lower Gonial angle), Ja12 (Jarabak’s ratio), Mc7 (Lower anterior facial height), Mc3 (Linear distance from point A to nasion perpendicular). In particular, the first 3 variables are the measurements that account for the direction of vertical growth of the mandible, which is the main determinant in the pattern of facial growth and it is therefore logical that they appear as the most important. On the other hand, the Mc3 variable is indicative of the sagittal position of the maxilla with respect to the skull, which is not considered a determinant of the pattern of facial growth, however it could be related, since the rotation of the mandible generally in normally, it is accompanied by a rotation of the maxilla in the same direction and magnitude.

In the case of Fig. 3, it is observed that Mc3 (Linear distance from point A to nasion perpendicular) is the parent node of Ja4 (Lower Gonial angle), however, there is no direct biological explanation to explain this relationship since Mc3 is a sagittal measurement of the maxilla and Ja4 a vertical measurement of the mandible. In turn, Ja4 is the parent node of the variables Ja12 (Jarabak’s ratio) and Mc7 (Lower anterior facial height), which can be explained because the three variables correspond to measures indicative of vertical growth, so that when increasing or decreasing a of them, the others also increase or decrease respectively proportionally.

## Conclusion

In this paper, we have presented an alternative learning method based on an evolution strategy to learn the weights for constructing the TAN classifier. We applied this method to the facial biotype classification problem, obtaining high accuracy values compared to other competitive classifiers. When analyzing a resulting BN from $(\mu ,\lambda )$-TAN, many of the interactions shown in the network had an orthodontic interpretation, nevertheless, there were a few which did not have a satisfactory biological explanation. Future research will consider more benchmark datasets as well as other medical applications.

## Methods

### Dataset description

We use the [27] dataset, which comprises 182 lateral teleradiographies taken from patients in Chile. 31 continuous attributes that describe the craniofacial morphology were computed for each one using cephalometric analysis (see Table 7). Orthodontists have personally classified and validated each lateral teleradiograph into one of the three categories (Brachyfacial, Dolichofacial, and Mesofacial).

**Table 7:** A description of the attributes [27]

Attribute	Description
Mc3	Linear distance from point A to nasion perpendicular
Mc5	Mandibular length (Condylion to Gnathion)
Mc6	Maxillary length (Condylion to Point A)
Mc7	Lower anterior facial height (Anterior nasal spine to menton)
St1	SNA angle (Sella-Nasion-A)
Ja1	Saddle angle (Nasion-Sella-Articulare)
Ja2	Articular angle (Sella-Articulare-Gonion)
Ja3	Upper Gonial angle (Articulare-Gonion-Nasion)
Ja4	Lower Gonial angle (Nasion-Gonion-Menton)
Ja5	Anterior cranial base length (Sella to Nasion)
Ja6	Posterior cranial base length (Sella to Articulare)
Ja7	Ramus height (Articulate to Gonion)
Ja8	Mandibular corpus length (Gonion to Gnathion)
Ja9	Cranial base and Mandibular length ratio (Sella-Nasion/Gonion-Gnathion)
Ja10	Posterior facial height (Sella to Gonion)
Ja11	Anterior facial height (Nasion to Menton)
Ja12	Jarabak’s ratio (Posterior facial height/Anterior facial height)
Ja13	Posterior cranial base and ramus height ratio (Sella-Articulare/Articulare-Gonion)
Ri9	Maxillary height angle (Nasion-Center of Face-A)
Ri10	Maxillary depth angle (Porion-Orbitale and Nasion-A)
Ri11	Palatal plane angle (Porion-Orbitale/anterior nasal spine-posterior nasal spine)
Ri12	Cranial deflection (Basion-Nasion/Porion-Orbitale)
Ri13	Anterior Cranial length (Center of Cranium to Nasion)
Ri15	Mandibular corpus axis (point Xi to point protuberance menti or Pm)
Ri16	Articular cavity position: Porion to Ptv (intersection of the distal outline of pterigomaxillary fissure perpendicular to the porion-orbitale plane)
Ri17	Mandibular ramus position (Porion-Orbitale/Center of Face-point Xi)
Ri18	Posterior height (Gonion to Center of Face)
Ri19	Condylar height
Ri20	Condylar neck length
Ri21	Symphysis length
Ar5	Nasolabial angle (Columella-Subnasale-upper lip)

### Alternative learning algorithm for tree augmented Naive Bayes

We propose an evolution strategy (ES) for learning TAN classifiers. The standard versions of the ES are denoted by [30]

2 $$\begin{aligned} (\mu ,\lambda )-ES \quad \text {and} \quad (\mu +\lambda )-ES, \end{aligned}$$

where $\lambda$ represents the number of offspring and $\mu$ the number of parents. From the multi-set of either the offspring, known as comma-selection ($\mu <\lambda$ must hold), or both the parents and offspring, known as plus-selection, the parents are deterministically chosen (i.e., deterministic survivor selection). Selection is based on the ranking of the individuals’ fitness, taking the $\mu$ best individuals (also referred to as truncation selection).

In this study, we generate weights for the TAN model that produce good facial biotype classification results without estimating the conditional mutual information by using the deterministic survivor selection $(\mu ,\lambda )$ technique.

In order to do this, a candidate solution (an individual) is encoded as an *m*-dimensional vector that holds the *m* weight values of a network. We must define $m=n(n-1)/2$ weights for a network of *n* nodes. Consequently, we must locate 465 weights for $n=31$. (parameters). We proceed as follows in order to determine the right values for these weights.

**Fig. 4:**
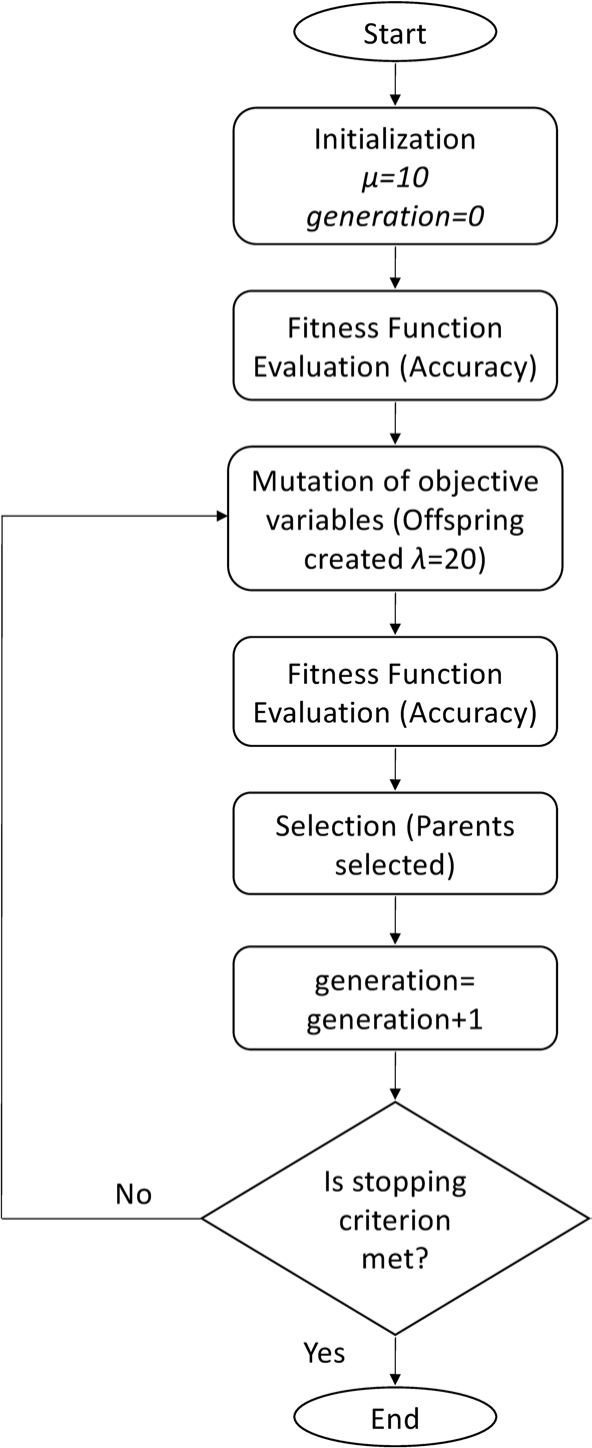
Alternative learning algorithm for tree augmented Naive Bayes. Flowchart of the evolution strategy

By evenly distributing all the weight values in each individual’s unit hypercube at random, we create an initial population of $\mu$ individuals. The procedure is then repeated a specified number of times. Each iteration starts with a population-wide evaluation of each proposed solution. A flowchart that briefly explains how the ES algorithm functions is shown in Fig. 4. The reader is directed to [31] for further information on how ES functions.

The accuracy, or the percentage of correctly classified instances in the training set, is then used to calculate the fitness function.

The highest scoring $\mu$ parents are then chosen. We perform the subsequent actions:

- The parent population consists of $\mu =10$ individuals.
- The number of offspring produced per each iteration is indicated by $\lambda =20$.
- Individuals die out after one iteration step (we use 1000 iterations) and only the offspring (the youngest individuals) survive to the following generation. Then, $\mu$ parents are chosen from $\lambda$ offspring via environmental selection.

These hyperparameters were selected through simulations of trial and error.

### Model performance assessment

We evaluated four metrics: precision (*Prc*), recall (*Rec*), accuracy (*Acc*), and $F_{1}$-score. This is how these measurements are calculated:

3 $$\begin{aligned} Prc= & {} \frac{TP}{TP+FP}\times 100 \end{aligned}$$

4 $$\begin{aligned} Rec= & {} \frac{TP}{TP+FN}\times 100 \end{aligned}$$

5 $$\begin{aligned} Acc= & {} \frac{\hbox {number of correctly classified instances}}{\hbox {total number of instances}}\times 100 \end{aligned}$$

6 $$\begin{aligned} F_{1}\hbox {-score}= & {} 2\times \frac{Prc\times Rec}{Prc+Rec} \end{aligned}$$

where *TP*, *TN*, *FP*, and *FN* stand for true positive, true negative, false positive, and false negative, respectively. Since we are dealing with a multiclass problem, we compute *Prc*, *Rec*, and $F_{1}\hbox {-score}$ for each individual class, and then report the average.

Additionally, we calculate the Kappa statistic, which contrasts the trained model’s Acc (in the test set) with a random model’s accuracy. We utilize the classification suggested in [32] to interpret the Kappa value: values $\le 0$ indicate poor agreement, $0-0.2$ indicate slight, $0.21-0.4$ indicate fair, $0.41-0.6$ indicate moderate, $0.61-0.8$ indicate substantial, and $0.81-1$ indicate practically perfect agreement.

### Experimental setup

The continuous features were discretized using Fayyad and Irani’s Minimum Description Length method [33], which has been shown to have a positive effect on the classifiers’ performance [34]. We compared the performance of the Naive Bayesian network classifier (NB), TAN, support vector machine (SVM) [35], decision tree (DT) [36], Random Forest (RF) [37], random vector functional link neural network [38] (RVFL), Averaged TAN (ATAN) [39] and the proposed method $(\mu ,\lambda )$-TAN. Four greedy hill-climbing algorithms were also used as a basis for learning Bayesian network classifiers:

- Hill-climbing tree augmented Naive Bayes (HC-TAN) [40].
- Hill-climbing super-parent tree augmented Naive Bayes (HC-SP-TAN) [40].
- Backward sequential elimination and joining (BSEJ) [18].
- Forward sequential selection and joining (FSSJ) [18].

The HC-TAN and HC-SP-TAN algorithms begin with a Naive Bayes structure and continue to add edges until the network score does not increase. Beginning with a Naive Bayes structure, BSEJ adds augmenting edges before removing features from the model until there is no longer any increase in the network score. On the other hand, FSSJ begins with a structure that only has the class node and builds upon it by adding features and enhancing edges.

Averages and standard deviations were recorded after 20 times of doing each experiment run. We divided the dataset into 70% for training and 30% for testing for each run, with the division being done at random.

We partitioned the original training set into 70% for training and the remaining 30% to evaluate different hyperparameter configurations through grid search for all the algorithms that needed hyperparameter tuning.

The open-source R software environment for statistical computation was used for all of the simulations. We used the test set’s kappa statistic and accuracy metric to assess classification performance. Additionally a statistical significance test, the paired sample t-test, for different simulations in terms of accuracy was conducted.

## Data Availability

The corresponding author can be contacted via email for direct access to the dataset used in this study.

## Abbreviations

BN: Bayesian networks
ES: Evolution strategy
NB: Naive Bayesian network classifier
TAN: Tree augmented Naive Bayes
SVM: Support vector machine
DT: Decision tree
RVFL: Random vector functional link neural network
ATAN: Averaged tree augmented Naive Bayes
$(\mu ,\lambda )$-TAN: Evolution strategy deterministic survivor selection tree augmented Naive Bayes
HC-TAN: Hill-climbing tree augmented Naive Bayes
HC-SP-TAN: Hill-climbing super-parent tree augmented Naive Bayes
BSEJ: Backward sequential elimination and joining
FSSJ: Forward sequential selection and joining
